# Multi-species Microbial Modulation of the RANK/RANKL (Receptor Activator of Nuclear Factor κB/Receptor Activator of Nuclear Factor κB Ligand) Pathway Induces Osteoclastogenic Priming in Apical Periodontitis

**DOI:** 10.7759/cureus.107487

**Published:** 2026-04-21

**Authors:** Victoria Alejandra Gutiérrez-López, Rita Elizabeth Martínez-Martínez, José Manuel Alderete-Hernández, Emmanuel López-González, Roberto González-Amaro, Bruno Rivas-Santiago, Selene Velázquez-Moreno, Marlen Vitales-Noyola

**Affiliations:** 1 Endodontics Postgraduate Program, Faculty of Dentistry, Autonomous University of San Luis Potosi, San Luis Potosí, MEX; 2 Master Program in Advanced Dentistry, Faculty of Dentistry, Autonomous University of San Luis Potosi, San Luis Potosí, MEX; 3 Posgraduate Program in Farmaceutical-Biological Sciences, Faculty of Chemical Sciences, Autonomous University of San Luis Potosi, San Luis Potosí, MEX; 4 Department of Immunology, Faculty of Medicine, Autonomous University of San Luis Potosi, San Luis Potosí, MEX; 5 Medical Research Unit, Mexican Institute of Social Security Delegation Zacatecas, Zacatecas, MEX; 6 Endodontics Postgraduate Program, Faculty of Dentistry, Autonomous University of San Luis Potosi, San Luis Potosi, MEX

**Keywords:** alveolar bone resorption, apical periodontitis, microorganisms, osteoclasts, rank and rankl

## Abstract

Background: Apical periodontitis (AP) is a prevalent inflammatory condition affecting the periapical tissues surrounding the tooth root, often initiated by microbial invasion.

Objective: The objective of this study is to evaluate the osteoclastogenic priming effects of microbial species associated with primary and secondary AP on human macrophages and to determine their capacity to modulate the RANK/RANKL-OPG (receptor activator of nuclear factor kappa-B, receptor activator of nuclear factor kappa-B ligand, osteoprotegerin) pathway​.

Materials and methods: THP-1 monocytes/macrophages were stimulated with live bacteria, with or without RANKL, and analyzed for RANK/RANKL/OPG expression by flow cytometry. Cytokine levels (interleukin-1β (IL-1β), tumoral necrosis factor-α (TNF-α), interleukin-6 (IL-6), interleukin-8 (IL-8), interleukin-10 (IL-10), interleukin-12p70 (IL-12p70)) were measured using cytometric bead arrays.

Results: Macrophages challenged with *Enterococcus*, *Streptococcus*, *Actinomyces*, and *Candida *exhibited an increase in RANK⁺, RANKL⁺, and RANK⁺RANKL⁺ cellular populations. *Enterococcus* and *Candida* promoted sustained RANK/RANKL expression across conditions, while *Streptococcus* and *Actinomyces* elicited significant double-positive cell expansion. Early microbial stimulation triggered robust TNF-α, IL-1β, and IL-6 production, peaking with *Streptococcus intermedius*, *Enterococcus faecalis*, and *Actinomyces*, followed by a decrease at 96 hours. In contrast, IL-8 secretion remained high among species and time points, with *Actinomyces* inducing the strongest response.

Conclusion: Our data suggest that AP-associated bacteria can induce osteoclastogenic priming and promote the release of pro-inflammatory cytokines via RANK/RANKL activation, indicating a potential role in the early processes leading to periapical bone resorption.

## Introduction

Apical periodontitis (AP) is a prevalent inflammatory disease of the periapical tissues caused by microbial infection within the dental pulp and root canal system. It is characterized by localized bone resorption, tissue destruction, and chronic inflammation [1]. Globally, up to 52% of adults are estimated to have at least one tooth affected by AP [2]. Maintenance of a healthy bone microenvironment depends on a tightly regulated balance between osteoclast-mediated bone resorption and osteoblast-mediated bone formation. Although AP is a localized infection, pathogens, their products, and inflammatory cytokines from periapical lesions can elicit systemic immune responses that compromise overall health [1]. The etiological agents of AP are diverse, including anaerobic microorganisms such as *Fusobacterium spp., Prevotella spp., and Porphyromonas spp*.; facultative anaerobic Gram-positive cocci such as *Enterococcus spp., Streptococcus spp., *and* Aerococcus spp.*; filamentous bacteria such as *Actinomyces spp*.; and opportunistic fungi such as *Candida spp*. [3-6]. Among these, *Enterococcus* is the most frequently isolated genus from infected root canals (38.5%), whereas *Candida albicans*, although detected in only 8% of cases, is notable for its persistence in endodontic infections and their resistance to standard eradication approaches [6]. Each group possesses distinct virulence factors that can shape the local inflammatory milieu and influence bone resorption. We have previously shown that *Enterococcus spp*. and *Streptococcus spp*. can drive osteoclast differentiation and stimulate the release of pro-inflammatory cytokines such as IL-6 and TNF-α, promoting disease progression [6]. *Actinomyces spp.* have been linked to heightened pro-inflammatory responses and immune cell skewing [7]. Molecular profiling further reveals that the microbial communities in AP are highly individual-specific, typically comprising only one to four predominant taxa, underscoring the heterogeneous etiology of the disease [8,9]. Through cell-wall components and secreted factors, these microorganisms can modulate macrophage-to-osteoclast differentiation and disrupt bone homeostasis.

Osteoclastogenesis is a tightly regulated process responsible for bone resorption, skeletal remodeling, and maintenance of structural integrity [10]. Macrophages and monocytes, central players in host defense, can differentiate into osteoclasts under the influence of microbial stimuli and host-derived factors [11]. This differentiation is orchestrated by the RANK/RANKL/OPG axis: Receptor activator of nuclear factor κB ligand (RANKL) binding to its receptor RANK promotes osteoclast differentiation, while osteoprotegerin (OPG) functions as a soluble decoy receptor that inhibits the process [12]. A shift in the RANKL/OPG ratio can therefore tip the balance toward pathological bone resorption, a hallmark of AP. Microbial and host factors converge to influence osteoclast differentiation. Pro-inflammatory cytokines, lipopolysaccharides (LPSs), and lipoteichoic acids (LTAs) are key mediators [13,14]. LPSs can induce osteoblasts to release RANKL and stimulate macrophages to produce TNF-α and IL-1 [15]. LTAs derived from *Enterococcus faecalis, Staphylococcus aureus*, and *Streptococcus pneumoniae *enhance inflammatory responses through TNF-α and IL-6, whereas LTAs from* Lactobacillus spp.* can promote anti-inflammatory pathways that inhibit osteoclastogenesis, likely dependent on LTA structure and D-alanine content [16,17]. Our previous work with staphylococcal LTA demonstrated a decreased RANK/RANKL ratio and an increased RANK/OPG ratio, suggesting that specific bacterial components can differentially modulate osteoclastogenesis [13]. Thus, the nature of the infecting microorganisms and their membrane-associated molecules determines whether bone resorption is promoted or suppressed.

In this context, the present study aims to address a critical gap in our understanding of AP pathogenesis by investigating how diverse microorganisms influence osteoclastogenic priming in macrophages. Specifically, we examine the capacity of these pathogens to modulate the RANK/RANKL-OPG pathway and trigger pro-inflammatory signaling. Clarifying these microbe-host interactions may inform novel therapeutic strategies to limit periapical bone resorption and preserve periapical health. By providing insight into the early molecular mechanisms that regulate bone remodeling in AP, this research contributes to a broader understanding of host-pathogen dynamics in chronic oral infections.

## Materials and methods

Samples 

Microorganism samples were obtained from infected root canals through paper tips or files, under aseptic conditions, following standard endodontic access procedures. Microorganisms isolated from these patients were subsequently incorporated into the institutional microbial strain collection for research purposes. In addition, a commensal strain of *Streptococcus salivarius* was isolated from the oral cavity of a healthy individual and included in the study to serve as a comparative control of oral commensal microorganisms. The research protocol was reviewed and approved by the Ethics and Research Committee of the Faculty of Dentistry, Universidad Autónoma de San Luis Potosí, under approval code CEIFE-021-021.

Isolation and identification of microorganisms 

All isolates obtained from clinical samples were initially cultured in thioglycolate broth and incubated under strict anaerobic conditions (85% N₂, 5% CO₂, 10% H₂) (COY Lab Products, Grass Lake, MI) at 37±2°C for 48-96 h or until visible growth. Growth density was monitored by turbidity measurements using a McFarland densitometer (Densimat, BioMérieux, Marcy-l'Étoile, France), and viable colonies were recovered on anaerobic CDC blood agar plates (BD Biosciences, San José, CA). Colony morphology and pigmentation were examined macroscopically, while microscopic features were assessed through stereoscopic observation and Gram staining to confirm bacterial classification. To ensure strain purity, subcultures were prepared, and biochemical characterization was carried out using semi-automated API Systems (BioMérieux, Marcy-l'Étoile, France) with interpretation through the API Web software. All isolates were compared against reference profiles to validate accuracy, and cryopreservation protocols were applied for long-term storage of confirmed strains. From the complete panel of microorganisms originally recovered, *Enterococcus faecalis, Enterococcus faecium, Actinomyces spp., Candida albicans, Streptococcus mitis, Streptococcus constellatus, Streptococcus intermedius, Streptococcus spp., *and* Streptococcus salivarius*, a representative subset was selected for experimental work. The choice was based on their recurrent detection in endodontic infections and their relevance in the pathogenesis of AP. Accordingly, the study focused on *Enterococcus faecalis, Actinomyces spp., Candida albicans, Streptococcus mitis, and Streptococcus constellatus* as the primary microorganisms for downstream assays, while *Streptococcus salivarius* was included as an oral microbiota commensal control.

Cell culture

The human monocyte leukemia cell line TPH-1 (Sigma-Aldrich, Darmstadt, Germany) was used in the assays related to cell differentiation induction. Briefly, 5.0X10^5^ cells were seeded in a 48-well plate format and cultured with RPMI-1640 media supplemented with 10% fetal bovine serum, 1% glutamine at 37±2°C and 5.0% CO_2_. Then, cells were stimulated with 50 ng/mL of phorbol myristate acetate (PMA, Sigma-Aldrich, Darmstadt, Germany) for five days to induce the differentiation into macrophages. Finally, the cells were washed and stained with 1% trypan blue. Although no functional assays for osteoclastogenesis (e.g., TRAP staining) were performed in this study, the differentiation of THP-1 cells into macrophages and subsequent osteoclastogenic priming was previously validated and extensively characterized in our earlier work [6]. Therefore, the methods used here follow the same standardized procedures, which have been confirmed in multiple independent experiments.

RANK expression in THP-1 cells under differentiation conditions 

To determine the role of microorganisms in inducing macrophage-to-osteoclast differentiation, 2.5 × 10⁵ cells/well were cultured in RPMI-1640 medium under three experimental conditions: (i) RANKL stimulation alone (1 μg/mL), which served as a positive control for osteoclast differentiation, (ii) microbial challenge alone, and (iii) combined RANKL and microbial challenge. The inclusion of the RANKL-only condition allowed us to confirm the responsiveness of THP-1-derived macrophages to osteoclastogenic signaling and provided a baseline for comparison with microbially induced effects. Microorganisms isolated from the root canals of study patients were added individually at a concentration equivalent to 0.5 on the McFarland scale. This concentration was previously standardized and validated in multiple experiments in our prior work [6] to ensure reproducibility and consistent multiplicity of infection (MOI) across different microbial species. Then, the co-culture was incubated at 37 ± 2°C in 5% CO₂ for two hours to allow microbial interaction with THP-1-derived macrophages. After this initial period, non-adherent microorganisms were removed by thorough washing to discard unbound bacteria, ensuring that only cell-associated microorganisms remained. The wells were then further incubated for 48 and 96 hours under the same conditions. During this extended incubation, extracellular bacterial outgrowth and intracellular survival were controlled through the washing steps and prior standardization of the initial microbial inoculum at 0.5 McFarland, which was previously validated in multiple experiments to ensure reproducible MOI across different microbial species [6]. After the incubation periods, cells were washed and cultured in the presence (experimental condition) or absence (negative control) of 50.0 ng/mL human recombinant RANKL (BioLegend, San Diego, CA). The cultures were maintained for up to seven days at 37±2°C with 5% CO₂, and analyses were performed at both 48 and 96 hours to evaluate RANK/RANKL expression and osteoclastogenic priming.

Flow cytometry 

Cells were harvested from culture wells and washed twice with phosphate-buffered saline (PBS) to remove residual media and non-adherent microorganisms. Cells were then resuspended in staining buffer (PBS supplemented with 1% bovine serum albumin (BSA)) prior to antibody labeling. For surface marker detection, cells were incubated with fluorochrome-conjugated monoclonal antibodies specific for human RANK (PE), RANKL (FITC), and OPG (APC) (BioLegend, San Diego, CA) for 30 minutes at 4°C in the dark to prevent fluorophore degradation and photobleaching. After incubation, cells were washed twice to remove unbound antibodies and resuspended in PBS with 1% BSA. To ensure data reliability and reproducibility, several controls were included in every experiment: single-stained samples and fluorescence minus one controls were used to set proper gating boundaries and correct for spectral overlap between fluorochromes. Compensation controls were prepared using antibody capture beads (CompBeads, BD Biosciences, San Jose, CA) stained individually with each fluorochrome, allowing accurate adjustment of emission spillover between detectors. Isotype-matched controls were incorporated to evaluate nonspecific binding and background fluorescence. Samples were acquired on a FACSCanto II flow cytometer (BD Biosciences) using standard instrument settings. For each sample, a minimum of 100,000 events were collected. Forward scatter (FSC) and side scatter (SSC) parameters were used to exclude debris, while doublets were removed using FSC-A/FSC-H gating. During acquisition, voltages and thresholds were optimized for each fluorochrome, and instrument performance was verified using calibration beads before each session. Data analysis was performed using FlowJo v10.0 software (Tree Star, San Diego, CA). Fluorescence compensation was applied using the automated matrix generated from single-stain controls. Subpopulations of interest, including RANK⁺, RANKL⁺, and OPG⁺ macrophages, were quantified as percentages of the total viable cell population. Results were confirmed in at least three independent experiments to ensure reproducibility, and representative gating strategies are illustrated in figure results. 

Quantification of inflammatory cytokines 

Supernatants from THP-1 macrophages, differentiated under the experimental conditions described above, were collected at 48 and 96 hours. The levels of pro- and anti-inflammatory cytokines, including IL-1β, TNF-α, IL-6, IL-8, IL-10, and IL-12p70, were measured using a cytometric bead array kit (BD Biosciences, San Diego, CA) according to the manufacturer’s instructions. Briefly, 50 µL of each supernatant was incubated with the fluorescent bead capture antibody mixture, followed by detection with phycoerythrin-conjugated secondary antibodies. Samples were protected from light and incubated at room temperature for the recommended time to ensure optimal binding. Flow cytometry acquisition was performed using an Accuri C6 flow cytometer (BD Biosciences, San Diego, CA) with standard voltage and threshold settings. A minimum of 5,000 events per bead population was collected to ensure statistical robustness. Bead populations corresponding to each cytokine were identified based on size and fluorescence, and data were analyzed using FCAP Array software v3.0 (BD Biosciences, San Diego, CA) for simultaneous quantification. Appropriate standards, single-bead controls, and assay blanks were included in each run to generate standard curves and to correct for nonspecific background. All measurements were performed in triplicate for each experimental condition, and results are presented as mean ± standard deviation. This approach ensured reproducibility and allowed for accurate comparisons of cytokine production between control and microbially stimulated THP-1 macrophages, reflecting the inflammatory and osteoclastogenic priming responses.

Statistical analysis

The data are displayed as the arithmetic mean, SD, median, and interquartile range (Q1-Q3 or IQR). Differences between two groups were analyzed with the t-student or Mann-Whitney U test, and comparisons among three or more groups were analyzed with one-way ANOVA or the Kruskal-Wallis sum-rank test, according to normal or non-normal distribution, respectively. For experiments evaluating effects over multiple time points, repeated measures ANOVA was applied to account for within-subject variability. When significant differences were observed, post hoc analyses were performed using Tukey’s (for ANOVA and repeated measures ANOVA) and Dunn’s (for Kruskal-Wallis) multiple comparison tests. Data were analyzed using the GraphPad Prism v5.0 software (GraphPad, San Diego, CA), and p-values <0.05 were considered significant.

## Results

Expression of RANK and RANKL in macrophages challenged with *Enterococcus, Actinomyces, *and* Candida albicans*

**Figure 1:**
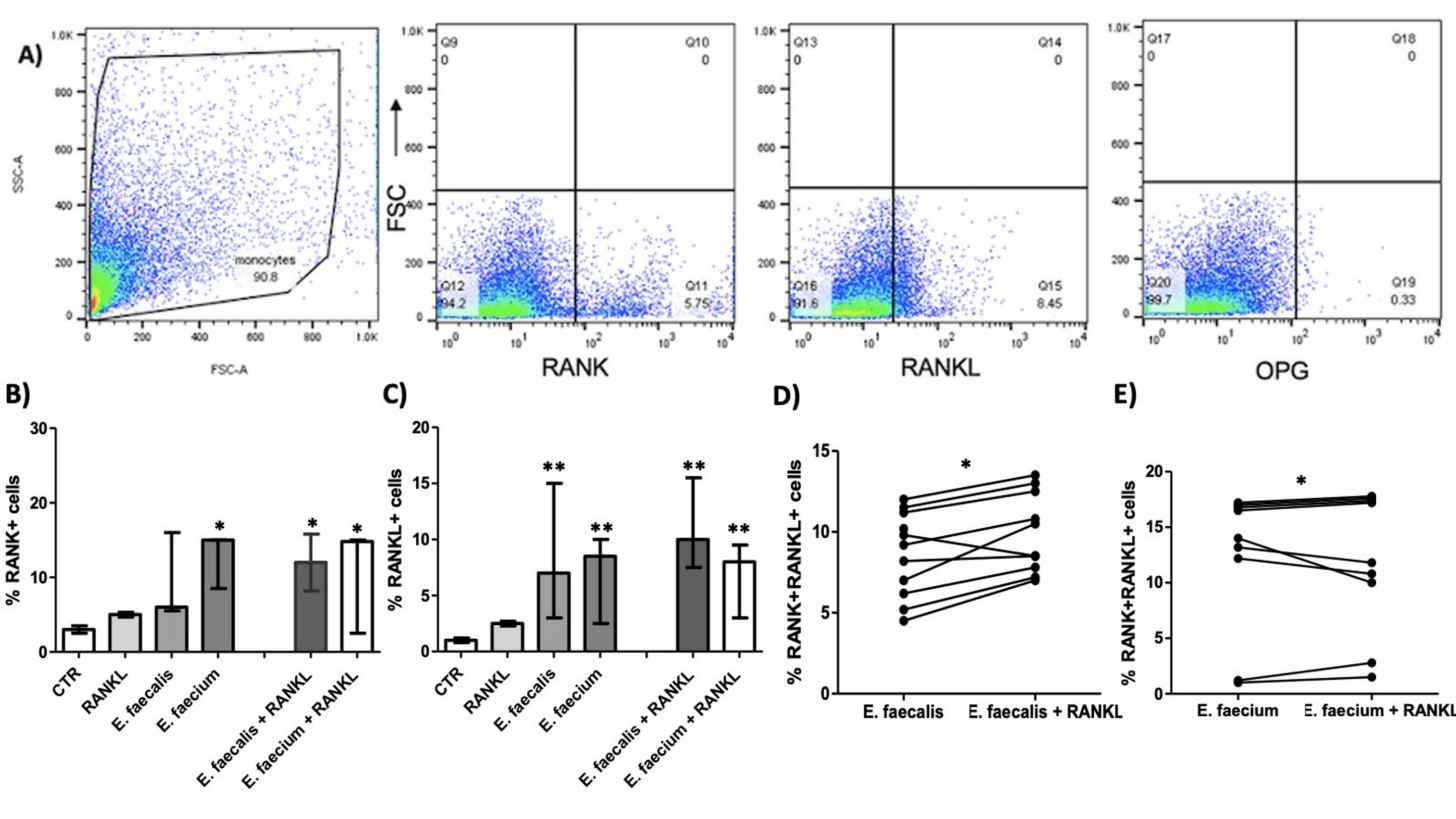
RANK and RANKL expression in monocyte-derived cells exposed to E. faecalis and E. faecium. The expression of RANK, RANKL, and OPG was evaluated in THP-1-derived macrophages following microbial stimulation in the presence or absence of soluble RANKL at 48h exposition, as stated in Materials and Methods. A) Representative dot plots of flow cytometry analysis showing gating strategy and expression of RANK, RANKL, and OPG in monocyte-derived cells exposed to *Candida albicans*. B) Percentage of RANK⁺ cells in macrophages treated with *E. faecalis* or *E. faecium*, in the absence or presence of RANKL. C) Percentage of RANKL⁺ cells under the same conditions. D) Paired comparison of the percentage of RANK⁺RANKL⁺ double-positive cells in cells exposed to different E. faecalis strains with or without RANKL. E) Paired comparison of RANK⁺RANKL⁺ cells exposed to different *E. faecium* strains with or without RANKL. Horizontal bars represent the median and the interquartile range. *p<0.05, **p<0.01 by paired or unpaired t-test. RANK: Receptor Activator of Nuclear Factor-κB; RANKL: Receptor Activator of Nuclear Factor-κB Ligand; OPG: Osteoprotegerin; CTR: control; *E. faecalis*: *Enterococcus faecalis*; *E. faecium*: *Enterococcus faecium*

Representative images of the flow cytometry strategy gating for RANK, RANKL, and OPG expression in *Candida albicans* at 48 hours are shown in Figure 1A.

**Figure 2:**
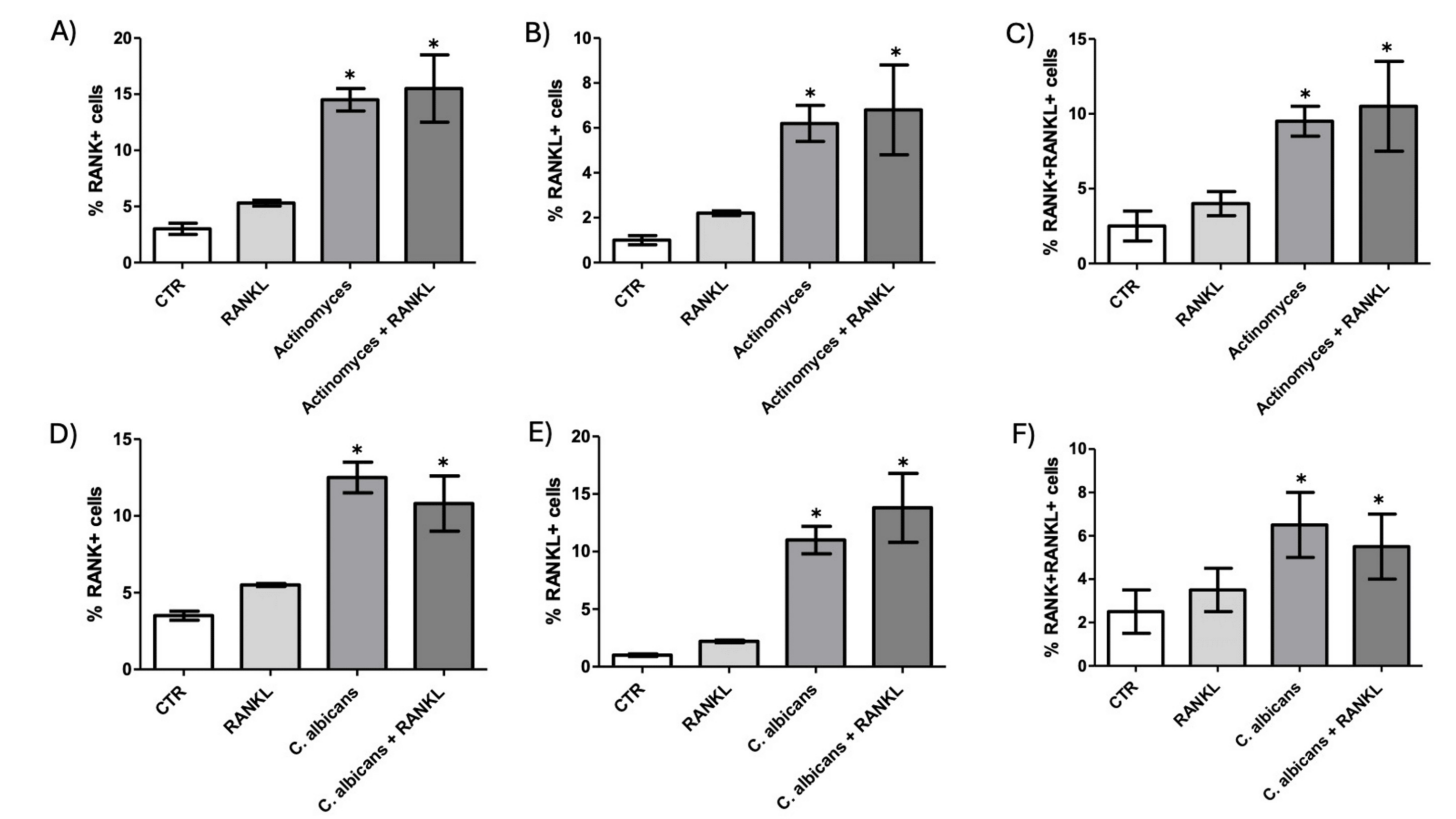
Actinomyces spp. and C. albicans promote osteoclastogenic priming in monocyte-derived macrophages. The expression of RANK and RANKL was evaluated in macrophages stimulated with Actinomyces spp. (A–C) or Candida albicans (D–F), with or without soluble RANKL supplementation for 48 hours. A, D) Percentage of RANK⁺ cells. B, E) Percentage of RANKL⁺ cells. C, F) Percentage of RANK⁺RANKL⁺double-positive cells. Both microbial stimuli significantly increased the expression of osteoclastogenic markers compared to the control, particularly in the presence of RANKL. Data are presented as mean ± SEM. *p<0.05 by one-way ANOVA with post hoc testing. RANKL: Receptor Activator of Nuclear Factor-κB Ligand; CTR: control; *C. albicans*: *Candida albicans*; RANK: Receptor Activator of Nuclear Factor-κB

According to this analysis, we found an increase of RANK^+ ^cells in macrophages stimulated with *E. faecium *in the absence of RANKL stimuli and an increase in both *E. faecalis* and *E. faecium* with RANKL stimuli (Figure 1B, p=0.05). Upon analysis, the RANKL+ macrophages in *E. faecalis* and *E. faecium* showed increased proportions in both conditions, independent of RANKL stimuli (Figure 1C, p=0.0047). On analyzing the pathogenicity of different strains of *E. faecalis* and *E. faecium* to modify the RANK+/RANKL+ cells, we observed a slight increase from almost all the strains in both microorganisms (Figures 1D, 1E, p=0.012 in both cases). We found that both *Actinomyces* and *Actinomyces* with RANKL stimuli increased the proportions of RANK+, RANKL+, and RANK+/RANKL+ cells in macrophages (Figures 2A-2C, p=0.015, p=0.016, and p=0.016, respectively).

*Candida albicans* showed the same results (Figures 2D-2F, p=0.012, p=0.015, and p=0.045, respectively). We found no changes in OPG+ cells or ratios for any of the studied microorganisms.

Expression of RANK and RANKL in macrophages challenged with *Enterococcus, Candida albicans, Streptococcus, Actinomyces*, and *Aerococcus*

**Figure 3:**
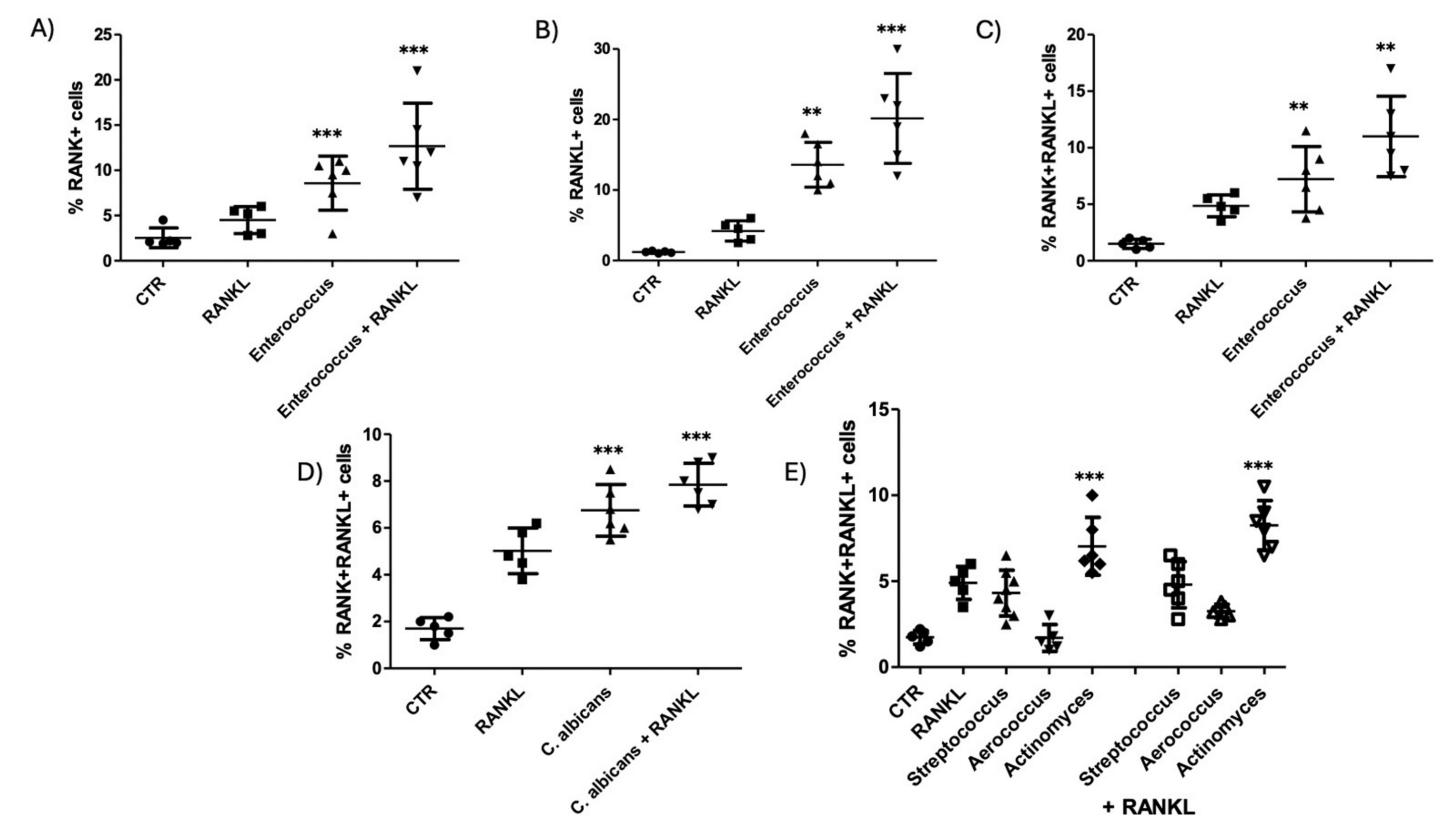
Differential modulation of RANK/RANKL expression by microbial species in monocyte-derived macrophages. Flow cytometry analysis was performed to quantify the percentage of macrophages expressing RANK⁺, RANKL⁺, and RANK⁺RANKL⁺ phenotypes following stimulation with various microbial species for 96 hours, with or without exogenous RANKL A–C) Enterococcus spp. Significantly increased RANK⁺ (A), RANKL⁺ (B), and RANK⁺RANKL⁺ (C) cells, especially in combination with RANKL. D) C. albicans significantly enhanced the percentage of RANK⁺RANKL⁺ cells under both conditions. E) Comparative analysis of RANK⁺ and RANKL⁺ expression following exposure to *Streptococcus* I., *Actinomyces* *spp*., and *Aerococcus* *spp*. in the presence of RANKL revealed significantly higher induction by *Streptococcus *and *Actinomyces*. Data are presented as mean ± SEM. *p<0.05, **p<0.01, ***p<0.005 by one-way ANOVA with post hoc testing. RANKL: Receptor Activator of Nuclear Factor-κB Ligand; CTR: control; *C. albicans*: *Candida albicans*; RANK: RANK: Receptor Activator of Nuclear Factor-κB

To further explore the osteoclastogenic potential of microbial species commonly associated with AP, we analyzed the percentage of RANK⁺, RANKL⁺, and double-positive RANK⁺RANKL⁺ macrophages in response to microbial exposure at 96 hours. Exposure to *Enterococcus spp.* led to a significant increase in the percentage of RANK⁺ (Figure 3A, p=0.0006), RANKL⁺ (Figure 3B, p=0.0001), and RANK⁺RANKL⁺ (Figure 3C, p=0.0001) macrophages, particularly in the presence of RANKL.

Similarly, *C. albicans *significantly elevated the frequency of RANK⁺RANKL⁺ cells regardless of RANKL stimulation (Figure 3D, p<0.0001). When comparing multiple bacterial genera, we found that both *Streptococcus spp.* and *Actinomyces spp*. Significantly increased the percentage of RANK⁺RANKL⁺ macrophages compared to control (Figure 3E, p<0.0001), whereas *Aerococcus spp*. failed to induce comparable changes under the same conditions. 

Proinflammatory cytokine production in macrophages exposed to *Streptococcus* and *Enterococcus*

**Figure 4:**
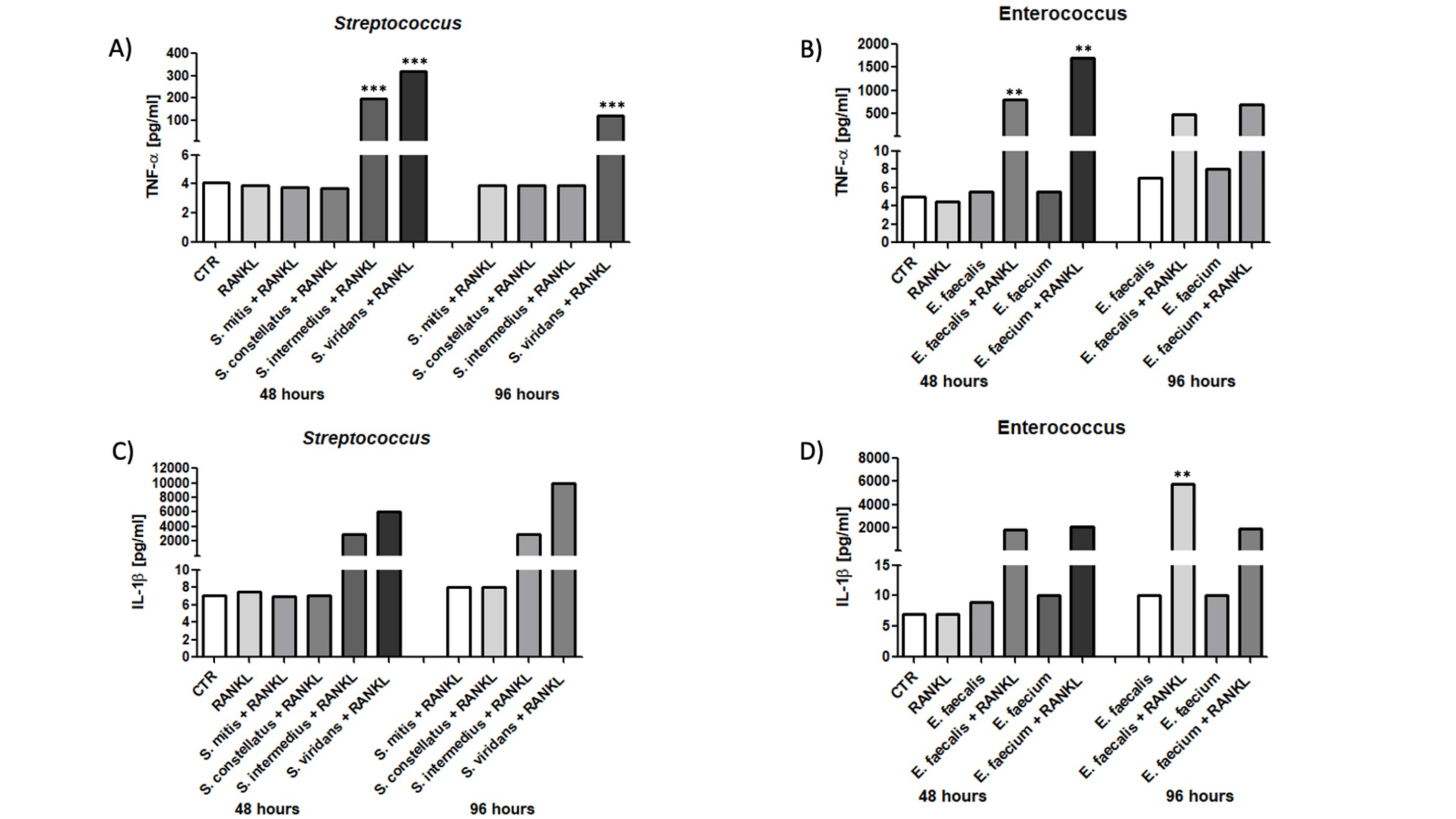
Proinflammatory cytokine production in macrophages exposed to Streptococcus and Enterococcus species. Cytokine levels were quantified in the supernatants of THP-1-derived macrophages stimulated with Streptococcus spp. or Enterococcus spp., either with or without RANKL, 48 and 96 hours post-stimulation. ELISA was used to measure concentrations of TNF-α (A, B), IL-1β (C, D), IL-6 (E, F), and IL-8 (G, H). A–D) TNF-α and IL-1β production increased significantly after 48 h of exposure to both bacterial genera, particularly with *S. mitis* and *E. faecalis*, and was further enhanced in co-treatment with RANKL.  Data are presented as mean ± SEM. *p<0.05, **p<0.01, ***p<0.005 by two-way ANOVA with multiple comparisons. RANKL: Receptor Activator of Nuclear Factor-κB Ligand; CTR: control; *S. mitis*: *Streptococcus mitis*; *S. constellatus*: *Streptococcus constellatus*; *S. intermedius*: *Streptococcus intermedius*.

To further characterize the inflammatory response associated with osteoclastogenic activation, we quantified proinflammatory cytokine production in macrophage supernatants at 48 and 96 hours following microbial challenge, as described in material and methods. Figure 4 summarizes the effects of microbial stimulation on RANK, RANKL, and OPG expression in THP-1-derived macrophages under different experimental conditions and time points. Stimulation with *Streptococcus spp.* led to a significant increase in TNF-α and IL-1β levels at 48 hours, with a marked decline by 96 hours (Figures 4A, 4C, p=0.0001 and p=0.0001, respectively)

**Figure 5:**
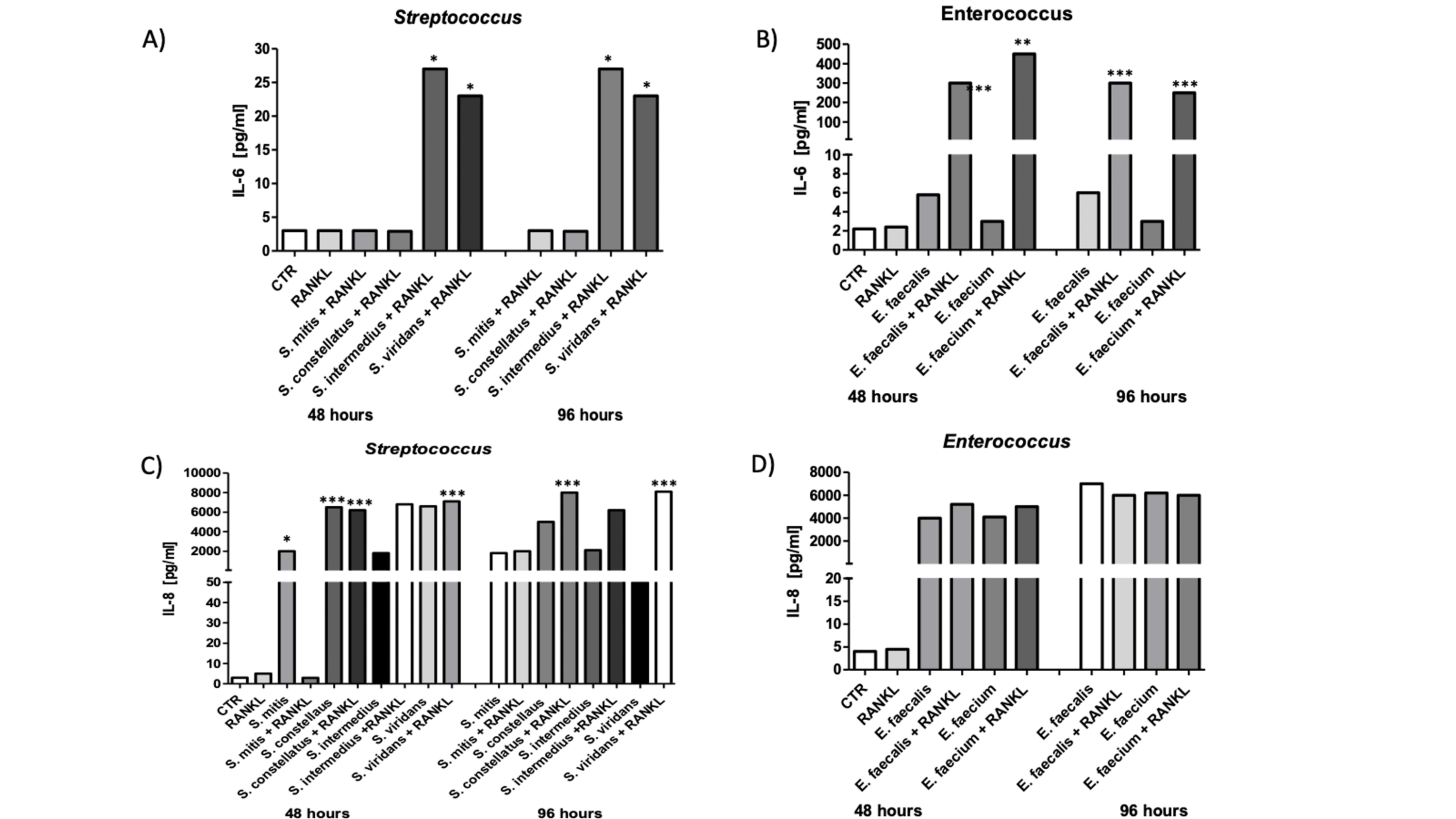
Proinflammatory cytokine production in macrophages exposed to Streptococcus and Enterococcus species. A-B) IL-6 levels showed a marked increase at 48 h in *S. intermedius* and *E. faecalis* groups, diminishing at 96 h. C, D) IL-8 secretion was consistently elevated in all microbial conditions across both time points. Split Y-axes were implemented to accommodate the large differences in scale between baseline control levels and peak cytokine production. *p<0.05, **p<0.01, ***p<0.005. *S. intermedius*: *Streptococcus intermedius*; *E. faecalis*: *Enterococcus faecalis*.

A similar temporal pattern was observed for *Enterococcus spp*., where TNF-α and IL-1β levels peaked at 48 hours and decreased by 96 hours, regardless of RANKL supplementation (Fig. 4B and 4D, p=0.047 and p<0.05, respectively). Regarding IL-6, exposure to *S. intermedius* induced a strong cytokine release at 48 and 96 hours, while *S. viridans* had a late effect increasing at 96 hours (Figure 5A, p=0.012), while *E. faecalis *and* E. faecium* also showed elevated IL-6 production, particularly in the presence of RANKL (Figure 5B, p=<0.0001). In contrast, IL-8 secretion was consistently high across all conditions and time points for both *Streptococcus* and *Enterococcus spp*., with minimal variation between 48 and 96 hours (Figures 5C, 5D, p<0.0001 and p=0.0065, respectively). 

Inflammatory cytokine response in macrophages stimulated with *A. viridans, Actinomyces spp*., and *C. albicans* at 48 and 96 hours

**Figure 6:**
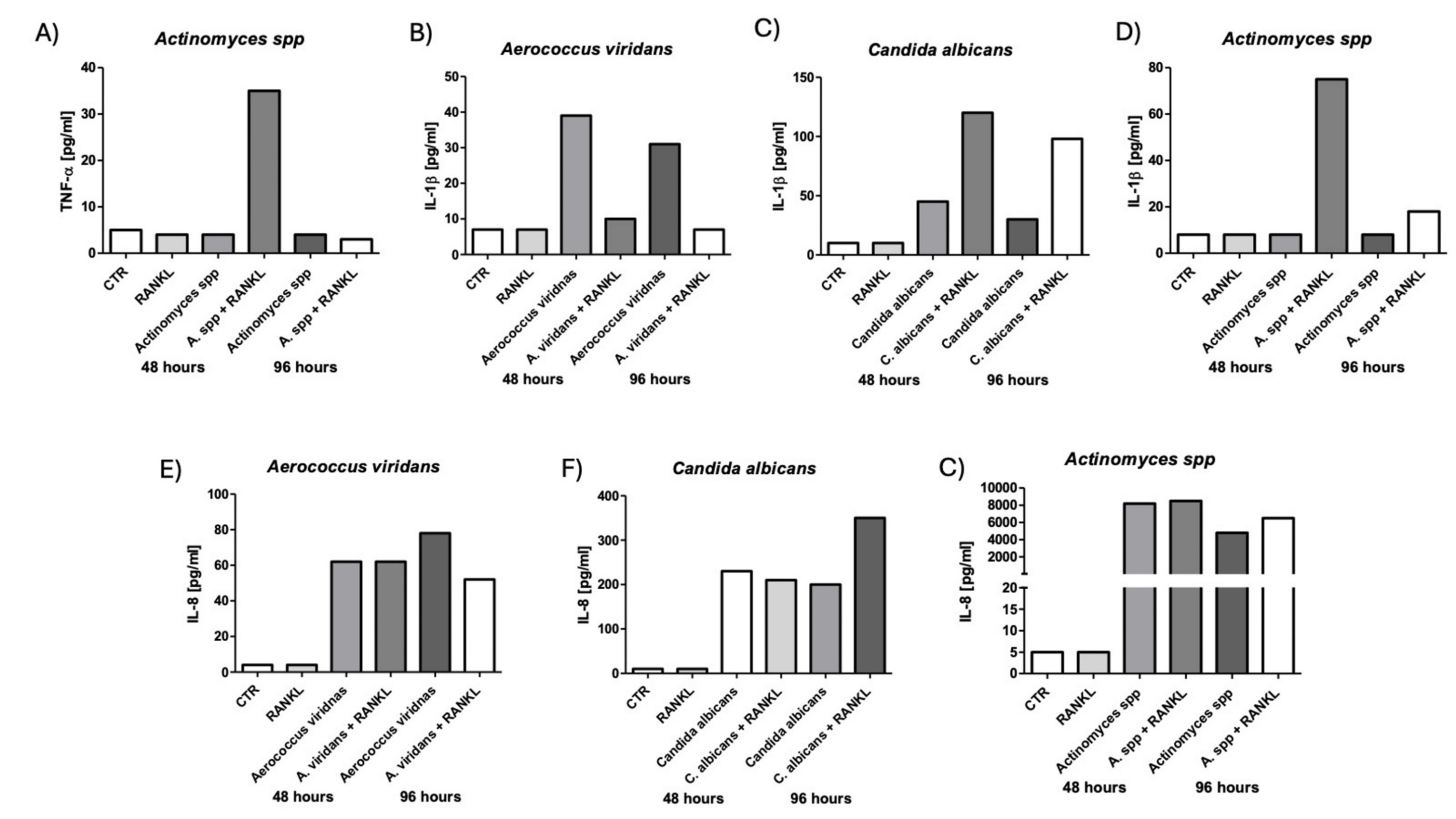
Cytokine production in macrophages stimulated with Aerococcus viridans, Actinomyces spp., and Candida albicans. Supernatants from macrophage cultures were harvested at 48 and 96 hours following microbial challenge, with or without RANKL supplementation. Cytokine concentrations were quantified by flow cytometry (A) TNF-α levels induced by *Actinomyces* spp. (B–D) IL-1β levels following exposure to *A. viridans*, *C. albicans*, and* Actinomyces spp.*, respectively. (E–G) IL-8 secretion in response to *A. viridans*,* C. albicans*, *and Actinomyces spp.*, respectively. Bars represent mean ± SD. *p < 0.05, **p < 0.01, ***p < 0.001. *A. viridans*: *Aerococcus viridans*; A. *spp*:* Actinomyces spp*; *C. albicans*: *Candida albicans*.

To further investigate the inflammatory potential of other pathogens involved in AP, we assessed the secretion of pro-inflammatory cytokines by macrophages challenged with *A. viridans, Actinomyces spp.*, and *C. albicans*, with or without RANKL co-stimulation, at 48 and 96 hours. TNF-α levels significantly increased in macrophages stimulated with *Actinomyces spp*. and RANKL at 48 hours, but returned to baseline levels at 96 hours (Figure 6A, p=0.001).

IL-1β levels also increased following stimulation with *A. viridans* and *C. albicans*, especially in the presence of RANKL (Figures 6B, 6C, p=0.0102 and p<0.05. A strong IL-1β response was similarly observed with *Actinomyces spp*., at 48 hours, which markedly decreased by 96 hours (Figure 6D, p<0.05). Regarding IL-8, stimulation with *A. viridans, C. albicans*, and *Actinomyces spp*. led to robust cytokine production at 48 hours, which was maintained or further elevated by 96 hours (Figures 6E-6G, p=0.006, p=0.0053, and p=0.0054). The strongest IL-8 induction was observed with *Actinomyces spp*., exceeding 8000 pg/mL, independent of RANKL co-stimulation (Figure 6G).

## Discussion

Several studies have identified *E. faecalis *as the predominant microorganism in AP, highlighting its role in bone destruction and persistent inflammation during disease progression [18]. However, our previous findings show that other microorganisms frequently co-occur in both primary (PAP) and secondary apical periodontitis (SAP) and also exert significant osteoclastogenic effects [6].

This broader microbial spectrum, which includes *E. faecium, Streptococcus spp., Actinomyces spp., Candida albicans, *and* Aerococcus viridans*, is often underappreciated in periapical bone loss. Therefore, this study investigated how endodontic pathogens influence the RANK signaling pathway in THP-1 macrophage differentiation to osteoclasts. Our findings indicate that all tested pathogens are capable of inducing osteoclastogenic priming in macrophages, likely through distinct and species-specific mechanisms. These results suggest that targeted therapeutic interventions may need to account for multiple microbial contributions without implying full osteoclast differentiation. We assessed the osteoclastogenic potential of key pathogens in PAP and SAP, including *E. faecalis, E. faecium, Streptococcus spp., Actinomyces spp., *and* C. albicans.* Notably, *E. faecalis* is detected in up to 77% of SAP cases [6,18]. Macrophage-to-osteoclast differentiation was evaluated via RANK pathway markers. Microbial exposure significantly increased the proportion of RANK⁺ and RANKL⁺ cells, with species-specific differences and a marked enhancement at 96 h. The findings suggest that extended stimulation encourages macrophages to prime for osteoclastogenesis, consistent with bone resorption evident in chronic AP in vivo. Interestingly, previous studies reported osteoclastogenesis using heat-killed *E. faecalis* (HKEF); our model, using live bacteria, yielded increased osteoclastogenic responses [19]. This divergence may stem from methodological differences, particularly the shorter 72 h exposure reported by Park et al. [19], or from the inactivation process, which could denature key virulence factors. Our findings, consistent with reports of attenuated effects for HKEF compared with live bacteria [20], underscore the complexity of host-pathogen interactions beyond individual bacterial components. Among the tested species, *Streptococcus spp.* elicited one of the most robust osteoclastogenic responses, in agreement with *in vivo* data showing that *S. gordonii *enhances bone resorption by increasing osteoclastogenesis and suppressing osteoblast activity [21]. Importantly, this is the first study to show that* C. albicans* and *Actinomyces spp*. can induce osteoclastogenic priming in AP, underscoring their potential roles as overlooked contributors to periapical bone pathology. In *C. albicans*, the mannan component has been shown to impair osteogenic differentiation in human dental pulp cells, suggesting a broader capacity to disrupt bone homeostasis beyond osteoclast activation [22]. Despite apparent upregulation of RANK and RANKL expression, OPG levels remained unchanged. Given OPG's function as a decoy receptor that binds RANKL to block RANK-mediated osteoclastogenesis, this stability suggests that inflammatory conditions in AP may impair counter-regulatory mechanisms that typically limit bone resorption.

The results in proinflammatory and anti-inflammatory cytokines showed elevated levels of TNF-α, IL-1β, IL-6, and IL-8, but unchanged IL-10 levels after microbial challenge. These cytokines drive osteoclastogenesis: TNF-α directly induces TRAP⁺ osteoclast RANKL-independent and increases RANK expression on precursors; IL-1 and TNF-α synergistically promote osteoclast activation; IL-6 targets osteoclasts and T/B lymphocytes; and IL-8 contributes to inflammatory cell recruitment and matrix degradation [23-25]. This profile supports a feed-forward loop of inflammation and bone resorption in AP lesions. In addition, a lack of IL-10 upregulation may worsen this imbalance, as IL-10 limits inflammation and osteoclastogenesis. Early-phase (48-96 h) dynamics contrast with Torres et al., who saw no TNF-α or IL-1β increase at seven days, suggesting that prolonged exposure induces regulatory or exhaustion effects [6].

From a clinical perspective, these findings indicate that AP is sustained not only by microbial persistence but also by a failure to mount adequate anti-osteoclastogenic counter-regulation. Conventional treatment: chemo-mechanical debridement, intracanal medication, and antimicrobial irrigation primarily aim to reduce microbial load [26,27], but do not directly address the inflammatory pathways driving bone resorption. Targeting the RANK/RANKL axis or downstream effectors has been proposed as a host-modulation strategy in other osteolytic conditions. Yet, its applicability in the polymicrobial environment of AP remains untested. Our data suggest adjunctive approaches aimed at modulating host responses could provide therapeutic benefit, particularly in refractory or complicated cases. Potential interventions include RANKL inhibition, blockade of downstream osteoclastogenic signaling pathways such as IRAK-4 inhibition to disrupt IL-1/TLR-mediated activation, or targeting terminal effectors like cathepsin K to prevent matrix degradation [13,28,29]. Evaluating these strategies in models that replicate the complex microbial ecology of AP will be essential to determine their translational potential.

This study has several limitations that should be considered when interpreting the results. First, the *in vitro* model cannot fully reproduce the complex periapical environment, including tissue architecture, immune cell diversity, and biofilm dynamics, which may affect the cellular response. Second, only a limited selection of microorganisms associated with AP was tested, and the exposure times (48-96 hours) reflect early responses and may not capture longer-term effects or later stages of osteoclastogenic priming. Third, the use of a single macrophage cell line (THP-1) may not fully replicate the behavior of primary human macrophages, although differentiation and priming conditions were previously validated in multiple experiments [6]. Additional methodological considerations include the standardization of the initial infectious dose (0.5 McFarland) and control of bacterial proliferation, which, while previously optimized, still limit the precise replication of *in vivo* multiplicity of infection. Although no functional osteoclastogenesis assays (e.g., TRAP staining or dentin resorption) were performed in this study, previous experiments conducted by our group with *E. faecalis* and *E. faecium* extensively validated the TRAP methodology, confirming osteoclast differentiation under similar conditions. Therefore, conclusions here are interpreted strictly in the context of cellular priming rather than definitive osteoclastogenesis. Finally, while *Streptococcus salivarius* was included as an oral commensal control, broader microbial diversity and *in vivo* validation would be necessary to fully extrapolate these findings to clinical AP.

## Conclusions

In conclusion, this study demonstrates that key AP-associated microbial species can induce osteoclastogenic priming in macrophages and modulate pro-inflammatory signaling, reflecting early mechanisms relevant to periapical bone resorption. The viability of bacteria, duration of exposure, and interspecies variability emerged as important determinants of host responses. These findings provide a biological basis for further investigation of targeted interventions aimed at modulating the RANK/RANKL signaling pathway or its upstream microbial inducers, which may contribute to improved management of persistent endodontic infections.
